# Re-decontamination of liquid mycobacterial cultures: Additional *Mycobacterium tuberculosis* yield in the era of Xpert MTB/RIF Ultra in Cape Town, South Africa

**DOI:** 10.4102/ajlm.v10i1.1529

**Published:** 2021-12-10

**Authors:** Dawood da Costa, Pieter Nel

**Affiliations:** 1 Division of Medical Microbiology and Immunology, Department of Pathology, Faculty of Medical and Health Sciences, Stellenbosch University, Cape Town, South Africa; 2 National Health Laboratory Service, Tygerberg Hospital, Cape Town, South Africa

**Keywords:** tuberculosis culture, liquid Mycobacterial Growth Indicator Tube culture, decontamination, re-decontamination, Xpert MTB/RIF Ultra

## Abstract

A retrospective review of liquid mycobacterial cultures was performed at a laboratory in South Africa from 01 January 2018 to 31 December 2018 to assess the increased yield in detecting *Mycobacterium tuberculosis* complex following sample re-decontamination. Only 9 of 99 (9%) re-decontaminated samples were culture positive for *M. tuberculosis* complex. Xpert MTB/RIF Ultra, concurrently performed on 7 of the 9 samples, detected *M. tuberculosis* complex in all but 1 sample. Re-decontamination of non-sterile samples did not increase the *M. tuberculosis* complex yield enough to offset the financial costs and additional labour in a laboratory that utilises the Xpert MTB/RIF Ultra system as a first-line diagnostic modality.

## Introduction

Tuberculosis is caused by *Mycobacterium tuberculosis*, which belongs to a group of closely related, slow-growing mycobacteria collectively referred to as the *M. tuberculosis* complex. In 2018, there were an estimated 10 million cases of tuberculosis globally, with seven million cases notified and 1.45 million tuberculosis-related deaths^1^. South Africa, in the same year, had an estimated 301 000 new tuberculosis cases^1^.

Detection of *M. tuberculosis* complex by culture method remains the gold standard for confirming tuberculosis disease.^2^ It can detect as few as 10 *M. tuberculosis* complex colony-forming units (CFU) per mL in clinical specimens.^3^ Liquid culture increases the number of bacteriologically confirmed cases of tuberculosis by 20% – 30%, even when rapid, sensitive nucleic acid amplification tests such as the Xpert Ultra (Cepheid, Sunnyvale, California, United States)^3,4^ are used. Culture is advantageous as it allows for phenotypic drug susceptibility testing of the isolates. Processing of specimens for culture is, however, costly, labour intensive, expertise requiring, and time-consuming (requiring up to six weeks to process to completion) owing to the slow proliferation time of *M. tuberculosis* complex.^5,6,7^

The Xpert Ultra assay is a closed, cartridge-based, nucleic acid real-time polymerase chain reaction system that allows for the simultaneous detection of *M. tuberculosis* complex and rifampicin susceptibility in under 2 h. It can be performed directly on clinical samples and has a 5 CFU/mL – 25 CFU/mL limit of detection; but, it has a lower sensitivity than tuberculosis culture in samples from people living with HIV and on samples where no acid-fast bacilli (AFB) are visualised on microscopy (smear negative).^3,4^

Clinical specimens from non-sterile sites that are submitted to the mycobacteriology laboratory may be contaminated by other more rapid-growing bacteria.^5,8^ These specimens undergo a digestion-decontamination procedure as recommended by the World Health Organization. The N-acetyl-l-cysteine sodium hydroxide digestion-decontamination allows *M. tuberculosis* to be cultured in a liquid culture medium despite reducing the organism viability by between 20% and 30%.^3,6,8,9^ A proportion of cultures will however remain contaminated despite standard decontamination procedures. An acceptable rate for contaminated cultures in liquid media is 5% – 8%.^5,6,7,8,10^

Global recommendations for re-decontamination exist,^7^ but no published data on an acceptable compliance level for re-decontamination could be found. The World Health Organization recommends that re-decontamination of contaminated liquid culture be performed when the first of two submitted cultures is ‘positive for *M. tuberculosis* and contaminated’ and the specimen requires drug susceptibility testing, while the second culture is negative for *M. tuberculosis*, and if the mycobacterial protein antigen 64 result is indeterminate, owing to the presence of contaminants.

The aims and objectives of the study were to determine the contamination rate at the Tygerberg Hospital (TBH) mycobacteriology laboratory; assess the increase in *M. tuberculosis* complex yield following re-decontamination in samples that had undergone Xpert Ultra testing; to assess laboratory non-compliance rates with regard to the recommended sample re-decontamination protocol; and to perform a cost analysis of the re-decontamination of specimens.

## Methods

### Ethical considerations

Ethical approval for this laboratory-based study was obtained from the Health Research Ethics Committee, Stellenbosch University, South Africa, (project identification: 14939, HREC X20/04/016). Informed consent was waived by the Stellenbosch University ethics committee for this laboratory-based study. Data remained confidential throughout the study.

### Study setting

This study was conducted at South Africa’s National Health Laboratory Service (NHLS) Division of Medical Microbiology and Immunology mycobacteriology laboratory located in TBH.

Annually, the TBH mycobacteriology laboratory processes approximately 10 000 specimens for *M. tuberculosis* complex culture from TBH. All samples from non-sterile sites for *M. tuberculosis* complex culture undergo decontamination with 1.25% N-acetyl-l-cysteine sodium hydroxide and the decontaminated samples are processed according to the Becton-Dickinson Mycobacterial Growth Indicator Tube (MGIT) testing protocol.^5^ Specimen re-decontamination is performed on specimens from anatomical sites that are not easily obtainable or contaminated specimens that are microscopy positive for AFB. Easily obtainable anatomical specimens from non-sterile sites, such as urine and sputum specimens, are not re-decontaminated.^11,12^

### Study design and data analysis

A retrospective study was conducted to determine the number of *M. tuberculosis* complex cultures performed from 01 January 2018 to 31 December 2018. Data were electronically extracted from the NHLS central data warehouse into a Microsoft Excel (Microsoft Office 2016, Microsoft Corporation, Redmond, Washington, United States) datasheet. Results were anonymised and stratified according to culture status: negative, positive or contaminated. Contaminated samples underwent further analysis to determine eligibility for re-decontamination; compliance rates were calculated, and results were verified on the NHLS database, Trakcare webview (TrakCare Lab version L6.10, 2012, InterSystems Corporation, Cambridge, Massachusetts, United States).

## Results

A total of 9585 *M. tuberculosis* complex cultures were performed; 8049 (82.1%) were culture-negative, 912 (9.3%) were positive for *M. tuberculosis* complex, 31 (0.3%) were positive for non-tuberculous mycobacteria, and 593 (6.0%) were contaminated.

A total of 139 samples were assessed for re-decontamination, of which 99 (71%) were appropriately re-decontaminated, 37 (29%) were appropriately denied re-decontamination (due to having multiple samples incubating), and 3 (2.2%) were inappropriately denied re-decontamination.

Three samples were eligible for re-decontamination but did not undergo re-decontamination: two were sputum samples, in which AFB was observed on the contaminated culture and had Xpert Ultra testing which detected rifampicin-susceptible *M. tuberculosis* complex, and one was a bronchoalveolar lavage sample in which a second sample was contaminated. Non-compliance to re-decontamination was low, with 97.8% of samples correctly assessed for re-decontamination.

Of the 99 re-decontaminated samples, 75 (76%) were culture-negative, 5 (5%) contaminated, 10 (10%) positive for non-tuberculous mycobacteria and nine (9%) were positive for *M. tuberculosis* complex. Of 99 re-decontaminated samples, 89 samples were from anatomical sites not easily obtainable and 10 were samples that were microscopy positive for AFB.

Of the 10 contaminated samples that were microscopy positive for AFB and that underwent re-decontamination, all were sputum samples; non-tuberculosis mycobacteria were isolated from three samples and *M. tuberculosis* was isolated from seven. Only six of the seven samples that had *M. tuberculosis* complex isolated on culture underwent Xpert Ultra testing and were all positive for *M. tuberculosis* complex.

In summary, only 9 of 99 (9%) re-decontaminated samples were culture positive for *M. tuberculosis* complex. On Xpert Ultra testing, 6 of the 9 (67%) tested positive for *M. tuberculosis* complex, 1 (11%) tested negative for *M. tuberculosis* complex, and 2 (22%) did not undergo Xpert Ultra testing (but were smear positive for AFB on an auramine stain).

## Discussion

We found that 6% of all specimens undergoing mycobacterial culture at the TBH mycobacteriology laboratory were contaminated, which is in keeping with internationally accepted contamination standards of 5% – 8% in liquid media.^5,6,7,8,10^

Non-compliance to the recommended re-decontamination standard operating procedure was low at 2.2%. These three samples were not re-decontaminated as the Xpert Ultra had detected *M. tuberculosis* complex on two samples, and the third sample had additional specimens still incubating.

We found that of the seven re-decontaminated samples that were microscopy positive for AFB and culture positive for *M. tuberculosis* complex, six (86%) were also Xpert Ultra positive for *M. tuberculosis* complex. A diagnostic accuracy study of the Xpert Ultra by Dorman et al., with study participants from South Africa, found the sensitivity for smear-positive *M. tuberculosis* complex to be 99%^4^ suggesting that Xpert Ultra would likely have detected *M. tuberculosis* complex in the sample that was not tested.

In our setting, re-decontamination of samples that have undergone Xpert Ultra testing only yielded one additional *M. tuberculosis* complex isolate and although the sample size to identify the additional positive *M. tuberculosis* complex yield is small, to our knowledge this is the first study assessing the additional *M. tuberculosis* complex yield in re-decontaminated samples. The correlation between Xpert Ultra and *M. tuberculosis* complex positivity in re-decontaminated samples also reflects the excellent utility of Xpert Ultra testing as the initial diagnostic test in the South African national tuberculosis-testing algorithm. This finding is likely due to the Xpert Ultra’s lower *M. tuberculosis* complex detection limit of 5 CFU/mL – 25 CFU/mL compared to its predecessor Xpert MTB/RIF.^3^

Currently, the cost of re-decontamination and additional liquid MGIT culture at TBH NHLS amounts to R79.22 (South African rand; approximately, $5.00 United States dollars) per sample. When considering the low additional *M. tuberculosis* complex yield, added labour, and long turnaround time to final culture result, re-decontamination is not a cost-effective option in the setting where Xpert Ultra is used as the initial diagnostic test.

### Limitations

This was a laboratory-based study using routinely available data. The treatment status of patients who submitted samples could not be obtained, which may have impacted on *M. tuberculosis* complex yield following re-decontamination. Owing to the small sample size eligible for re-decontamination, and varying laboratory decontamination protocols, the findings in this study may not allow generalisation of our findings to other centres.

### Conclusion

The poor increase in yield of *M. tuberculosis* complex after re-decontamination of samples reflects the efficiency of the South Africa tuberculosis-testing algorithm, which employs Xpert Ultra testing, that has low limit of detection. In our high-burden tuberculosis setting, routine re-decontamination is not cost-effective and not recommended in specimens that have undergone Xpert Ultra testing.

## References

[1] World Health Organization. Global Tuberculosis Report. Geneva, Switzerland: WHO; 2019.

[2] Lewinsohn DM, Leonard MK, Lobue PA, et al. Official American Thoracic Society/Infectious Diseases Society of America/Centers for Disease Control and Prevention Clinical Practice Guidelines: Diagnosis of Tuberculosis in Adults and Children. Clin Infect Dis. 2017;64(2):e1–e33. https://doi/org/10.1093/cid/ciw694 27932390 10.1093/cid/ciw694

[3] Chakravorty S, Simmons AM, Rowneki M, et al. The new Xpert MTB/RIF ultra: Improving detection of Mycobacterium tuberculosis and resistance to Rifampin in an assay suitable for point-of-care testing. MBio. 2017;8(4):1–12. https://doi/org/10.1128/mBio.00812-17 10.1128/mBio.00812-17 PMC5574709 28851844

[4] Dorman SE, Schumacher SG, Alland D, et al. Xpert MTB/RIF Ultra for detection of Mycobacterium tuberculosis and rifampicin resistance: a prospective multicentre diagnostic accuracy study. Lancet Infect Dis. 2018;18(1):76–84. https://doi/org/10.1016/S1473-3099(17)30691-6 29198911 10.1016/S1473-3099(17)30691-6 PMC6168783

[5] Siddiqi SH, Rüsch-Gerdes S. MGIT Procedure Manual for Bactec MGIT960 TB System. Geneva, Switzerland: Foundation for Innovative New Diagnostics; 2006.

[6] Kent PT, Kubica GP. Public Health Mycobacteriology: A Guide For The Level III Laboratory. Atlanta, Georgia: Centre for Disease Control; 1985.

[7] Fitz-gerald M, Lumb R, WHO global TB programme. Laboratory safety – The handbook global edition [homepage on the Internet]. Geneva: GLI Working Group Secretariat; 2019. [cited 2020 Oct 23]. Available from: http://www.stoptb.org/wg/gli

[8] Garcia LS. Clinical Microbiology Procedures Handbook. Third Edit. (Garcia LS, ed.). Santa Monica, California: ASM Press; 2007.

[9] Mtafya B, Sabiiti W, Sabi I, et al. Molecular bacterial load assays concurs with culture on NaOH-induced loss of Mycobacterium tuberculosis viability. J Clin Microbiol. 57(7). https://doi/org/10.1093/jac/dku415 10.1128/JCM.01992-18 PMC6595441 31018981

[10] Stop TB Partnership (World Health Organization). Childhood TB Subgroup. Global Laboratory Initiative Advancing TB Diagnosis. Mycobacteriology Laboratory Manual. First edit. Geneva, Switzerland: Global Laboratory Initiative; 2014.

[11] Goodway J, Rautenbach C. Processing of positive MGIT vials: National Health Laboratory Service Tygerberg laboratory standard operating procedure MIC1223. Cape Town, South Africa: NHLS; 2019.

[12] Rautenbach C, Goodway J. TB Cultures: National Health Laboratory Service Tygerberg laboratory standard operating procedure MIC1222. Cape Town, South Africa: NHLS; 2019.

